# Screening and Identification of Host Proteins Interacting with *Iris lactea* var. *chinensis* Metallothionein IlMT2a by Yeast Two-Hybrid Assay

**DOI:** 10.3390/genes12040554

**Published:** 2021-04-10

**Authors:** Zhiquan Wang, Longjie Ni, Liangqin Liu, Haiyan Yuan, Suzhen Huang, Chunsun Gu

**Affiliations:** 1 Institute of Botany, Jiangsu Province and Chinese Academy of Sciences, Nanjing 210014, Jiangsu, China; zhiquanjiejie@163.com (Z.W.); liangqinliu100@126.com (L.L.); yuanhaiyan416@163.com (H.Y.); hsz1959@163.com (S.H.); 2 College of Forest Sciences, Nanjing Forestry University, Nanjing 210037, Jiangsu, China; longjie_ni@163.com; 3 Jiangsu Key Laboratory for the Research and Utilization of Plant Resources, Jiangsu Provincial Platform for Conservation and Utilization of Agricultural Germplasm, Nanjing 210014, Jiangsu, China

**Keywords:** *Iris lactea* var. *chinensis*, cadmium, metallothioneins, yeast two-hybrid, interaction

## Abstract

*Iris lactea* var. *chinensis* (Fisch.) (*I. lactea* var. *chinensis*) is a well-known cadmium (Cd)-tolerant plant and we have previously shown that the metallothionein gene, *IlMT2a*, of the plant may be playing a key role in conferring the Cd tolerance. In this study, we have identified several proteins interacting with the IlMT2a by screening yeast two-hybrid library constructed from cDNAs isolated from Cd-treated *I. lacteal* var. *chinensis* plants. Putative functions of these proteins include those involved in photosynthesis, ROS scavenge, nutrient transport, and transcriptional regulation, to name a few. In particular, another metallothionein, which we assigned the name of IlMT3, was identified as an interacting partner of the IlMT2a. Unlike IlMT2a, it did not provide any significant protection against Cd toxicity in transgenic *Arabidopsis thaliana* L. (*A. thaliana*). To our knowledge, this is the first time ever reporting the interaction of two metallothionein proteins in plants. Learning the biological significance of the interaction between IlMT2a and IlMT3 would be the focus of future study and would be able to provide valuable insights into the understanding plant metallothionein’s diverse and complex roles in coordinating many important cellular physiologies including stress responses, gene regulations, and energy metabolisms.

## 1. Introduction

In addition to facing conventional abiotic stresses, plants must contend with rapid environmental changes, which are due mainly to human activity and include soil and air pollution, climate change, etc. For example, heavy metals naturally exist in soils as rare elements, but factors such as refuse dumping, transportation, metal processing, and the use of Cd-containing phosphate fertilizers increase the amount and spread of Cd in the environment [1,2]. Furthermore, the increased use of recycled water, which contains heavy metals, for agricultural and horticultural irrigation leads to heavy metal contamination of soils [3,4]. Cd accumulation is not conducive to growth and development of plant [5,6]. Analysis of Cd tolerance-related genes is, therefore, significant to the development of plant germplasm resources in Cd-contaminated areas [4] and functional identification of plant resistance genes to Cd could accelerate the understanding of the molecular mechanisms that regulate tolerances to stresses [4].

*I. lactea* var. *chinensis* is a herbaceous perennial which has characteristics of fast growth, high ornamental value, large biomass, and wide adaptability. Research has demonstrated a strong tolerance to Cd in *I. lactea* var. *chinensis*, which indicates that it could be used as phytoremediation material for soils heavily contaminated with Cd [7,8]. An understanding of the physiological metabolism and molecular mechanism used by *I. lactea* var. *chinensis* under the pressure of Cd has gradually been developed [6,7,8].

Metallothioneins (MTs), which have a wide distribution in animals, plants, fungi and prokaryotes, are protein molecules that are rich in cysteine, have relatively low molecular weights, and contain metal-binding regions [9]. In order to reduce oxidative stress, MTs can also act as reactive oxygen species (ROS) scavengers. Plant MTs are classified into four types because of the type of cysteine residues [10]. Owing to high-affinity binding to heavy metals, the roles of plant MTs in detoxification of heavy metals and maintaining steady state of necessary metal ions in cell have been extensively researched [11,12,13]. *TcMT2a*, for instance, promotes the metal-adaptive phenotype [14]. While in *A. thaliana*, *BcMT2* helps to enhance Cd tolerance and reduce ROS production [15].

Previous studies have determined that *IlMT2a* (Genebank accession number AB907787), expression in *I. lactea* var. *chinensis* increases under Cd stress [7]. When constitutively expressed in *A. thaliana*, *IlMT2a* leads to a longer root length under Cd stress compared with the wild-type [7]. Histochemical staining has shown that the accumulation of H_2_O_2_ and O_2_^−^ in transgenic plants is significantly reduced compared with the wild-type [7]. Therefore, *IlMT2a* may be helpful in increasing plant tolerance to Cd. In wheat and rice, some transcription factors (TFs) confer Cd tolerance by increasing the expression levels of MT genes [16]. However, the mechanism of *IlMT2a* activity under Cd stress is yet unknown. A system of yeast two-hybrid was used in this research to screen proteins associated with IlMT2a from the cDNA of library of Cd-treated *I. lactea* var. *chinensis*. Notably, the function of the proteins is discussed. This study offers an experimental basis for studying the mechanism of Cd tolerance involving *IlMT2a*.

## 2. Materials and Methods

### 2.1. Plant Materials, Growth Conditions, and Treatment

*I. lactea* var. *chinensis* seedlings were adopted from the *Iris* Resource Collection Garden of Nanjing Sun Yat-Sen Memorial Botanical Garden and cultured in 1/2 Hoagland nutrient solution [17]. When seedlings reached a height of 10 cm, the plants were planted in 80 mg·L^−1^ CdCl_2_ [7]. After CdCl_2_ treatment, samples, including leaves and roots, were taken at 0, 1, 3, 6, 12, and 24 h, frozen rapidly in liquid nitrogen, and stored at −80 °C. The total sample (mixture combining all time point-treated plants) was collected for library construction.

### 2.2. cDNA Expression Library Construction and Quality Assay

The RNA from the total sample of *I. lactea* var. *chinensis* was extracted with a TRIzol RNA Kit (Invitrogen), the integrity and purity of the total RNA and purified mRNA were detected using both a nucleic acid analyzer (Thermo Nanodrop, (Thermo Scientific, Wilmington, DE, USA)) and 1% agarose gel electrophoresis. The total RNA above 300 µg with ratios of A260/A280 between 1.8 and 2.2 and concentration of total RNA above 150 ng/µL was chosen for subsequent analysis. The purification of mRNA above 1 µg was done with an Oligotex mRNA Midi Kit (QIAGEN, Hilden, Germany) for cDNA Library Construction.

A CloneMiner II cDNA Library Construction Kit (Thermo Fisher Scientific, Waltham, MA, USA) was used to construct the cDNA entry library. The process was as follows: reverse transcription using the primer Biotin-attB2-Oligo(dT), synthesis of double-stranded cDNA, fractionation followed by connection with an attB1-adapter, base pair reaction using a pDONR/222 vector and synthesis of cDNA followed by transformation into ElectroMAX™ DH10B™ through electroporation. Diluted (100-fold) library bacilli were cultured on LB solid medium (containing 100 mg·L^−1^ kanamycin) and the clones were counted after 12 h. Twenty-four clones were picked out for PCR detection with a universal primer pair for the pDONR/222 vector (F1/R1: GTAAAACGACGGCCAG/CAGGAAACAGCTATG AC).

A PureLink^®^96 Plasmid Purification System (Invitrogen, Carlsbad, CA, USA) was selected to isolate mixed plasmids and then the pGADT7 vectors (prey plasmids) were used to perform the LR gateway reaction. The transformations of reaction products into ElectroMAX™ DH10B™ competent cells were done for creating the yeast two-hybrid cDNA library. As above, a confirmation of the yeast two-hybrid cDNA library was performed with a universal PCR primer pair for the pGADT7 vector F2/R2: TAATACGACTCACTATAGG GCGAGCGCCGCCATG/GTGAACTTGCGGGGTTTTTCAGTATCTACGATT. The isolation of library plasmids were performed using the PureLink^®^96 Plasmid Purification System (Invitrogen) and subjected to yeast two-hybrid screening.

### 2.3. Bait Plasmid Construction

For the analysis of yeast two-hybrid, the cDNA sequences of *IlMT2a* (open-reading frame, ORF, GeneBank accession No. AB907787) were cloned and fused into the pGBKT7 vectors according to the manufacturer’s instructions (In-Fusion Advantage PCR Cloning Kit, Clontech, Mountain View, CA, USA). Sequencing was used to confirm the recombinant pGBKT7-*IlMT2a* plasmid (as the bait plasmid).

### 2.4. Assays of Auto-Activation and Toxicity of the Bait Plasmid

The Yeastmaker™ Yeast Transformation System 2 (Cat. No. 630439, Clontech, Mountain View, CA, USA) was adopted to respectively transform the pGBKT7-*IlMT2a* and pGBKT7 plasmids into Y2HGold. Transformants were incubated on SD/-Trp and SD/-Trp/X agar plates for 3–5 days at 30 °C. pGADT7-T and pGBKT7-Lam were co-transformed and grown on SD/–Leu/–Trp/X-a-Gal (DDO/X) plates (negative control) and pGADT7-T and pGBKT7-53 were co-transformed and grown on DDO/X (positive control). If the colonies containing pGBKT7-*IlMT2a* plasmid on SD/−Trp and SD/-Trp/X plates did not appear blue, it was confirmed that the bait had not auto-activated. If colonies containing the pGBKT7-*IlMT2a* plasmid were not smaller than those containing the pGBKT7 plasmid, the bait was verified as being non-toxic. Bait plasmids not showing auto-activation or toxicity were utilized in screening by the yeast-two-hybrid system.

### 2.5. Yeast-Two-Hybrid Screening with Co-Transformation

To screen out host proteins that have interaction with IlMT2a, the Yeastmaker™ Yeast Transformation System 2 was adopted to co-transform the prey plasmids and pGBKT7- *IlMT2a* into Y2HGold. The co-transformants were planted on SD/-Leu/-Trp/X-α-Gal/AbA (DDO/X/A) agar plates at 30 °C for 3–5 days. Colonies with blue were picked out and transferred to a higher stringency agar plate, as SD/-Ade/-His/-Leu/-Trp/X-α-Gal/AbA (QDO/X/A). To test the insertion size of each potential positive prey plasmid, PCR was operated using pGADT7-F/R primers (provided by Takara).

### 2.6. Confirmation of the Screened Interactions

In order to make confirmation of the screened interactions, pGBKT7-*IlMT2a* bait and each screened prey plasmid were again co-transformed into Y2HGold. The confirmation steps were as follows. An Easy Yeast Plasmid Isolation Kit (Cat. No. 630467, Clontech, CA, USA) was used to isolate each screened prey plasmid from putatively positive clones. To increase the concentration of plasmid, the isolated plasmid was transformed into DH5α and the TIANprep Yeast Plasmid DNA Kit (Cat. No. DP112, Tiangen, China) was used to purify plasmids from transformants growing on LB (containing 100 μg·μL^−1^ ampicillin) agar plates. Subsequently, each hypothetically positive prey plasmid and pGBKT7-*IlMT2a* bait plasmid were co-transformed into Y2HGold and grown on QDO/X/A plates to verify the interactions. Under these conditions, blue colonies exhibited true positive interactions.

### 2.7. Analysis of True Positive Prey

The confirmed true positive prey plasmids were sent for sequencing and online NCBI databases were used to BLAST the sequences and analyze the function of positive prey [18].

### 2.8. Functional Identification

The *A. thaliana* was cultured and used for functional identification of the confirmed proteins which can interact with IlMT2a. The *A. thaliana* ecotype Colombia (*Col-0*) was used as WT control. The deduced protein sequence of the confirmed interacting protein was analyzed by ClustalX and constructed phylogenetic tree with MEGA 7.0 using the Neighbor-Joining (NJ) method and 1000 bootstrap replicates. Then the coding sequence of cDNA was amplified and inserted into the Xba I sites of pCAMBIA1305 following digestion with the enzyme. This construct was transformed into *A. thaliana* using *Agrobacterium tumefaciens* strain EHA105 as described previously [7]. Seeds of transformed *A. thaliana* were selected using 1/2 MS medium supplemented with 20 mg/L hygromycin and 25 mg/L Kanamycin. Three homozygous lines of T3 generations were used for further analysis. For Cd stress tolerance assay, seeds were germinated and grown on 1/2 MS medium supplemented with 0, 25, 50, 100 μM CdCl_2_. The plates were placed in a vertical orientation upon onset of growth. After 10 d of growth, root lengths of transgenic lines and wild-type were measured. Statistical analysis was performed using SPSS and all data are represented as the means ± standard error. A double-sample equal variance hypothesis *t*-test was used to further analyze whether there were significant differences in the average value of root length. Differences were considered significant if *p* < 0.05 and *t*-test (*p* < 0.05).

## 3. Results

### 3.1. Construction of cDNA Library of I. lactea var. chinensis for Yeast Two-Hybrid Assays and pGBKT7-IlMT2a Bait Plasmid

The mRNA exhibited excellent quality (Supplement Figure S1A). The total yeast two-hybrid cDNA library included 1.2 × 10^7^ clones (Supplement Figure S1B) and the average inserted fragment length of 24 selected clones was above 1 kb (Supplement Figure S1C). The results suggested that the cDNA library was of acceptable quality and can be adopted for further assays by yeast-two-hybrid system. The concentration and quantity of the prey plasmids were 300 ng·µL^−1^ and 240 µg, respectively. The ORF of *IlMT2a*, which is 237 bp long, was amplified from the cDNA of *I. lactea* var. *chinensis*. The pGBKT7-*IlMT2a* bait plasmid was successfully constructed as confirmed by sequencing.

### 3.2. Assay Results of Auto-Activation and Toxicity of the Bait Plasmid

The transformation of pGBKT7-*IlMT2a* bait plasmid into Y2HGold was done and the transformants were cultured on SD/-Trp and SD/-Trp/X agar plates to detect any auto-activation activity. The colonies containing pGBKT7-*IlMT2a* were white on SD/-Trp and SD/-Trp/X plates and the colonies of the positive control were blue, indicating that pGBKT7-*IlMT2a* had no activity of auto-activation. The size of the Y2HGold colonies transformed by the bait plasmid were not smaller than the size of the Y2HGold colonies of the positive and negative controls. As a result, the pGBKT7-*IlMT2a* bait plasmid was qualified to be adopted in the next yeast-two-hybrid assay.

### 3.3. Yeast-Two-Hybrid Screening and Confirmation of the Interactions between pGBKT7-IlMT2a and Screened Prey Plasmids

**Figure 1:**
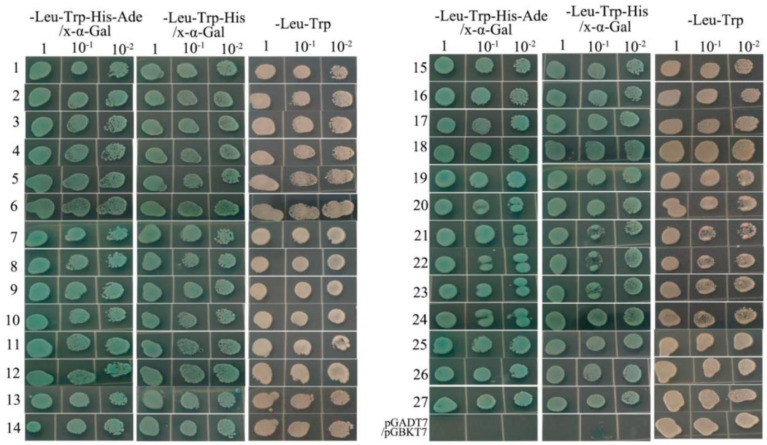
Interaction validation analyses of pGBKT7-*IlMT2a*.

The pGBKT7-*IlMT2a* was co-transformed with the prey plasmid and planted on DDO/X/A plates where 192 clones demonstrated a blue appearance (Supplement Figure S2). These 192 clones were subsequently transferred onto QDO/X/A plates. Fifty-nine of these 192 colonies still showed the color blue, suggesting that they may be positive (Supplement Figure S3). To exclude false positive clones, each of the 59 screened prey plasmids extracted from *E. coli* was co-transformed into Y2HGold cells with pGBKT7-*IlMT2a* and cultured on QDO/X/A plates. Based on these results, 27 host proteins were identified and confirmed to interact with *IlMT2a* (Figure 1).

### 3.4. Sequencing and Analysis of Confirmed True Positive Prey Plasmids

The 27 confirmed true prey plasmids were sent for sequencing with the pGADT7-F/R primers to determine sequence information for the confirmed true host proteins that have interaction with IlMT2a. The sequence results were analyzed with the online BLAST tool from the NCBI (Supplement Table S1).

### 3.5. Effects of IlMT3 on Cd Tolerance of A. thaliana

**Figure 2:**
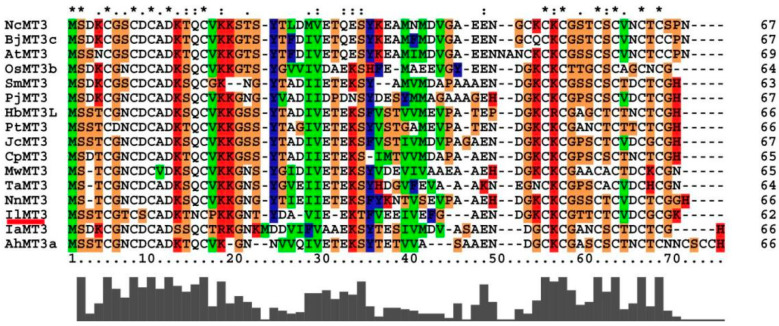
Multiple alignment of the IlMT3 amino acid sequence with sequences from different species. **Note:** NcMT3 (gi|125620188 [*Nelumbo nucifera*]), BjMT3c (gi|18958245 [*Brassica juncea*], AtMT3 (gi|18400732 [*Arabidopsis thaliana*]), OsMT3b (gi|158513348 [*Oryza sativa*]), SmMT3 (gi|351630004 [*Salvia miltiorrhiza*]), PjMT3 (gi|185178054 [*Prosopis juliflora*]), HbMT3L (gi|312985277 [*Hevea brasiliensis*]), PtMT3 (gi|62554179 [*Populus alba* x *Populus tremula* var. *glandulosa*]), JcMT3 (gi|282848222 [*Jatropha curcas*]), CpMT3 (gi|2497906 [*Carica papaya*]), MwMT3 (gi|2497907 [*Musa acuminata*], TaMT3 (gi|257219675 [*Typha angustifolia*]), NnMT3 (gi|125620188 [*Nelumbo nucifera*]), IaMT3 (gi|308083485 [*Ipomoea aquatica*]), AhMT3a (gi|110270325 [*Arachis hypogaea*]).

**Figure 3:**
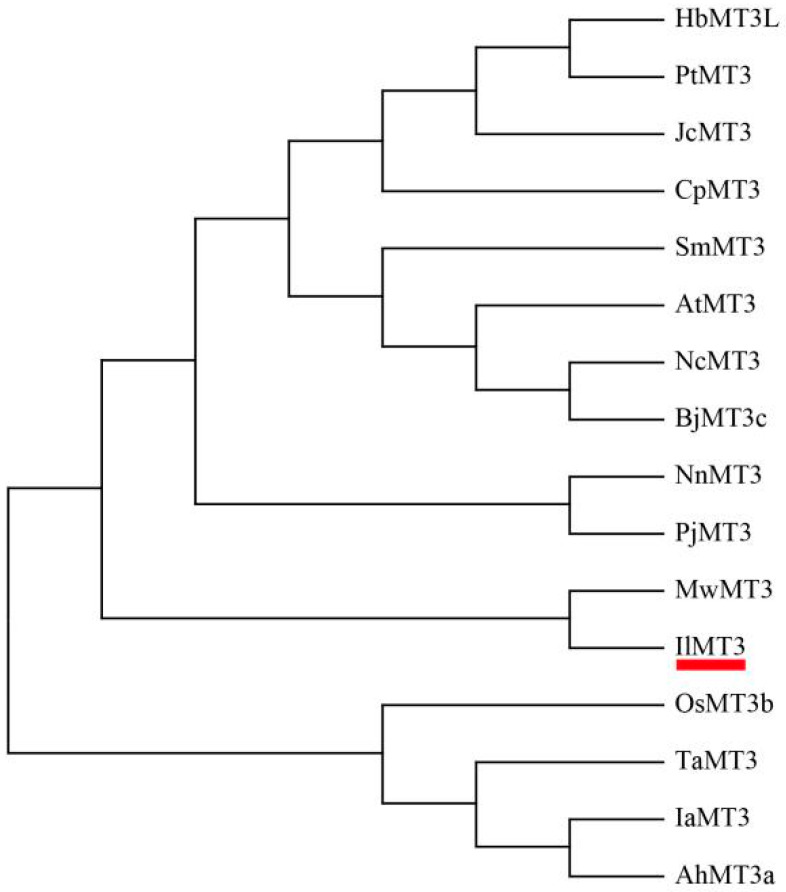
Phylogenetic analysis of IlMT3.

In this study, metallothionein (BAP25847.1) was screened out as an interact protein of IlMT2a. The amino acid sequence alignment indicated that the protein showed high homology to known MT3 proteins from other species, as a result, the protein was named as IlMT3 (Figure 2). The phylogenetic analysis showed that IlMT3 was closely related to MT3 proteins from *Musa acuminata* (Figure 3), which helped to reconfirm the identity of the protein. In order to identify the function of *IlMT3*, the full length gene cloned in pCAMBIA1305 vector was introduced into *A. thaliana* plants by *Agrobacterium*-mediated transformation (Supplement Figure S4). To evaluate Cd tolerance of transgenic *A. thaliana*, wild-type and 3 transgenic lines were transferred to 1/2 MS agar medium containing different concentrations of Cd to compare root elongation. Root elongation was severely inhibited in all plants under CdCl_2_ treatments. But there was no significant difference between the root lengths of transgenic lines and wild-type under control and CdCl_2_ treatments (*t*-test *p* < 0.05) (Table 1 and Supplement Table S2).

## 4. Discussion

MTs are widely distributed in plants and classified into four types based on their cysteine residues. Previous studies have demonstrated that in the Cd hyperaccumulator *Noccaea caerulescens*, *MT1* and *MT2* have higher levels of expression than in *A. thaliana*, indicating that *MT1* and *MT2* are significant to Cd tolerance [10]. In addition, *MT1* and *MT2* have the abilities of enhancing Cd tolerance of yeast or increasing the concentration of intracellular Cd [19,20]. It was also determined that the transcription factor can induce MTs to confer Cd tolerance [5,16]. For example, a heat shock transcription factor can enhance tolerance of wheat and rice to Cd by increasing the expression of *MT* genes [21]. Thus, functional studies of *IlMT2a* are necessary to better understand the mechanism of resistance to Cd in *I. lactea* var. *chinensis*. A yeast two-hybrid cDNA library was constructed in this study with Cd-treated *I. lactea* var. *chinensis*. A total of 27 host proteins were indicated to interact with IlMT2a, including MTs, ammonium transporter, photosystem II reaction center W protein (PSbW), polyubiquitin, NF-X1-type zinc finger protein (NFXL2), transcription factor bHLH, and AP2-like ethylene-responsive transcription factor TOE3.

One of the commonly found detoxification strategies found in the presence of Cd is the production of chelating compounds containing thiols, such as MTs, that interact strongly with Cd^2+^, reducing free Cd^2+^ in the cytosol, thereby limiting its toxicity [22,23]. Interestingly, another metallothionein was screened out in this study as an interact protein of IlMT2a. Based on the amino acid sequence alignment and phylogenetic analysis, it was named as IlMT3. While *IlMT3*-overexpressed transgenic *A. thaliana* plants did not exhibit enhanced Cd tolerance, which function is not similar to that of *IlMT2a* in previous study [7]. The *Tamarix hispida* metallothionein gene *ThMT3* increases tolerance against Cd in transgenic yeast, but it has not been shown *IlMT3* in *I. lactea* var. *chinensis* provide the same protection to plants in this study [24]. To our knowledge, this is the first time ever reporting the interaction of two metallothionein proteins in plants. The result indicated that *IlMT2a* can bond with Cd ions, forming non-toxic or low-toxicity compound, and thus as a main strategy to eliminate the toxicity of Cd, and also can cooperate with other members of MTs in *I. lactea* var. *chinensis* to perform different functions, which deserve to be further researched. As a result, learning the biological significance of the interaction between IlMT2a and IlMT3 would be the focus of future study and would be able to provide valuable insights into understanding plant metallothionein’s diverse and complex roles in coordinating many important cellular physiologies including stress responses, gene regulations, and energy metabolisms. Additionally, transport system proteins such as ATPases, ABC transporters and other heavy metal transporters have been found to improve resistance to heavy metals [22,25,26]. To reduce the toxic potential, transporters are located in the plasma or vacuolar membrane of plants and transport Cd^2+^ to apoplasts or vacuoles, respectively [22]. Following Cd^2+^ treatment, the expressive abundance of ammonium transporter *AtAMT1.1* was shown to increase 3–5-fold [27]. As a result, two detoxification strategies relatively related to MTs and ammonium transporters may exist and cooperate in *I. lactea* var. *chinensis*.

Damage to the photosynthetic machinery of plants under Cd stress produces a general decline in photosynthetic efficiency [2]. Cd destroys the photosynthetic organs, especially the two photosystems, leading to higher non-photochemical quenching [28]. The Photosystem II complex (PSII) is critical in regulating photosynthesis because it catalyzes the oxidation of water to oxygen and supports electron transport [29]. The complex is composed of a core, an oxygen evolving complex, and a light-collecting antenna system [29]. *PsbW* encodes a precursor to a polypeptide related to the reaction center of the PSII [30]. In a previous study, the expression of the unigeneen coding PsbW was substantially induced to mitigate Cd-induced damages in a low-Cd-accumulating cultivar of *Brassica parachinensis* [31]. According to the roles of *IlMT2a* in *I. lactea* var. *chinensis*, PSBW may similarly be involved in MT2a-PSBW interaction and this deserves further investigation.

NFX1-like proteins regulate the growth of plants through coordinating responses of ROS, SA, and ABA [32]. Distinctive structural features with the special C4HC3 RING finger motif and the conserved cysteine-rich region, along with the nuclear localization of proteins such as AtNFXL1 indicate that the E3 ubiquitin ligase-activity and bound DNA existed in NFX1-like proteins in plant [32]. However, deficiencies in R3H and PAM2 motifs in proteins of plant compared with those of other organisms indicate functional differences [32]. One of the major objectives in the functional description of plant NFX1-like proteins is the recognition of DNA-binding sites and screening for interacting proteins, as these may reveal regulatory mechanisms in stress responses [32]. *AtNFXL2*, a NFX1-like gene, is involved in the regulation responses to salt and osmotic stress in *Arabidopsis* [33]. Plants with impaired expression of *AtNFXL2* were similar to plants with increased expression of *AtNFXL1*, indicating that the *AtNFXL1* and *AtNFXL2* genes produce an antagonistic response under stress [33]. Previous reports have demonstrated that the contents of H_2_O_2_ and O_2_^−^ in *IlMT2a* transgenic lines are remarkably lower than those of the wild-type [7] and NFXL2 may play a key role in regulating the signaling pathway for metal-induced ROS. However, whether NFXL2 can regulate ubiquitin and how it interacts with IlMT2a in regulating the stress response still needs further study.

The regulation of TFs shows the complexity of the plant response to Cd stress [34]. Cd regulates the expression level of ERF proteins belonging to the AP2/EREBP family and DREB factors harbor an AP2/ERF domain that is important for its binding to DRE/CRT sequences in promoters of stress-inducible genes [5,35]. The bHLH family plays a key role in modulating plant cellular and physiological functions [36]. It has been demonstrated that AtbHLH38 and 39 could interact with AtbHLH29 to upregulate Cd-tolerance in *Arabidopsis* seedlings by reducing the transfer of Cd from roots to shoots, promoting homeostasis and building the concentration of iron in shoots [37]. In this study, AP2-like ethylene-responsive transcription factor TOE3 and bHLH130 as host proteins were indicated to interact with IlMT2a, suggesting that TFs may play key roles in raising Cd tolerance through interaction with IlMT2a.

## 5. Conclusions

In this study, several host proteins that interact with IlMT2a were screened out and identified. This is the first report of IlMT2-interacting proteins. The results suggested that IlMT2a may be involved in regulating photosynthesis, metal-induced ROS signaling pathways and transport systems. TFs belonging to different families may play key roles in regulating the expression of *IlMT2a* after Cd treatment. IlMT3, as an interacting protein of IlMT2a, cannot regulate Cd tolerance, indicating that IlMT2a can bond with Cd ions, forming non-toxic or low-toxicity compound, and thus as a main strategy to eliminate the toxicity of Cd, and also can cooperate with other members of MTs in *I. lactea* var. *chinensis* to perform different functions. These results provide better understanding of the functions of *IlMT2a* in Cd tolerance. Further study about IlMT2a should focus on the biological significance of the interaction between IlMT2a and IlMT3 and will provide valuable insights into understanding plant metallothionein’s diverse and complex roles in coordinating many important cellular physiologies including stress responses, gene regulations, and energy metabolisms.

## Figures and Tables

**Table 1:** Root lengths of 3 *IlMT3* lines and WT under control and Cd treatments (*p* < 0.05).

Treatments	Root Lengths of WT (cm)	Root Lengths of OE3 Lines (cm)	Root Lengths of OE4 Lines (cm)	Root Lengths of OE7 Lines (cm)
1/2 MS	3.23 ± 0.09 ab	3.43 ± 0.03 a	3.13 ± 0.12 b	3.43 ± 0.03 a
25 μM CdCl_2_	1.97 ± 0.07 d	2.10 ± 0.06 cd	2.17 ± 0.09 cd	2.27 ± 0.12 c
50 μM CdCl_2_	1.37 ± 0.07 e	1.50 ± 0.06 e	1.37 ± 0.07 e	1.47 ± 0.03 e
100 μM CdCl_2_	0.37 ± 0.03 f	0.53 ± 0.03 f	0.37 ± 0.03 f	0.40 ± 0.06 f

**Note:** Different lowercase letters indicate significant differences (*p* < 0.05).

## Data Availability

The data presented in this study are available in supplementary materials.

## Supplementary Materials

The following are available online at https://www.mdpi.com/article/10.3390/genes12040554/s1. Figure S1: Yeast-two-hybrid library of *I. lactea* var. *chinensis*. (A) Analysis of purified mRNA by 1% agarose gel electrophoresis; (B) titer of yeast-two-hybrid library; (C) inserted fragment detection of yeast-two-hybrid library. Figure S2: Co-transformation of pGBKT7-*IlMT2a* and 192 prey plasmids and growth on DDO/X/A plates. Figure S3: Co-transformation of pGBKT7-*IlMT2a* and 59 prey plasmids and growth on a higher stringency DQO/X/A plates. Figure S4: Semi-quantitative PCR result of *IlMT3* transgenic *A. thaliana*. Table S1: Analysis results of the confirmed true prey proteins identified using an online BLAST search. Table S2: The result of root lengths statistical analysis of t-test after Cd treatment.
