# Supplementary material for: Screening and Identification of Host Proteins Interacting with Iris lactea var. chinensis Metallothionein IlMT2a by Yeast Two-Hybrid Assay
Source: Genes (Basel). 2021 Apr 10;12(4):554. doi: 10.3390/genes12040554 (PMC8069374; doi:10.3390/genes12040554)
Supplement: Supplementary file 1 [file genes-12-00554-s001.zip › Supplement/Table S1.docx]

Table S1. Analysis results of the confirmed true prey proteins identified using an online BLAST search.

| **Gene Number** | **Predicted Protein** | **Accession Number** |
| --- | --- | --- |
| 1 | PREDICTED: photosystem II reaction center W protein, chloroplastic-like [Elaeis guineensis] | XP_010937454.1 |
| 2 | Protein ALTERED XYLOGLUCAN 4-like  [Asparagus officinalis] | XP_020267758.1 |
| 3 | 40S ribosomal protein S15-like isoform X1  [Asparagus officinalis] | XP_020256182.1 |
| 4 | Elongation factor 1-alpha  [Apostasia shenzhenica] | PKA56211.1 |
| 5 | U-box domain-containing protein, partial  [Trifolium pratense] | PNX98218.1 |
| 6 | PREDICTED: transcription factor bHLH130-like isoform X2 [Elaeis guineensis] | XP_010913124.1 |
| 7 | 40S ribosomal protein S20-1  [Phoenix dactylifera] | XP_008803986.1 |
| 8 | Uncharacterized protein LOC109828799  [Asparagus officinalis] | XP_020251332.1 |
| 9 | Metallothionein  [Iris lactea var. lactea] | BAP25847.1 |
| 10 | PREDICTED: protein LAZY 1 isoform X2  [Elaeis guineensis] | XP_010917537.1 |
| 11 | AP2-like ethylene-responsive transcription factor TOE3, partial [Asparagus officinalis] | XP_020271487.1 |
| 12 | 60S ribosomal protein L38  [Prunus persica] | XP_007202833.1 |
| 13 | PREDICTED: FAM10 family protein At4g22670  [Elaeis guineensis] | XP_010919047.2 |
| 14 | Uncharacterized protein A4U43_C05F32550  [Asparagus officinalis] | ONK70324.1 |
| 15 | NF-X1-type zinc finger protein NFXL2  [Phoenix dactylifera] | XP_008793296.1 |
| 16 | Hypothetical protein PHAVU_008G125700g  [Phaseolus vulgaris] | XP_007140597.1 |
| 17 | Exosome complex component RRP45A-like  [Asparagus officinalis] | XP_020253161.1 |
| 18 | Auxin-repressed 12.5 kDa protein  [Asparagus officinalis] | XP_020256719.1 |
| 19 | Hypothetical protein KP79_PYT21272  [Mizuhopecten yessoensis] | OWF52082.1 |
| 20 | Outer envelope pore protein 24, chloroplastic-like [Asparagus officinalis] | XP_020258018.1 |
| 21 | Palmitoyl-monogalactosyldiacylglycerol delta-7 desaturase, chloroplasticisoform X1  [Asparagus officinalis] | XP_020251329.1 |
| 22 | NF-X1-type zinc finger protein NFXL2 zf-NF-X1 [Asparagus officinalis] | XP_020266088.1 |
| 23 | Polyubiquitin (ubq10)  [Arabidopsis thaliana] | CAB81074.1 |
| 24 | Hypothetical protein B296_00045387  [Ensete ventricosum] | RRT34018.1 |
| 25 | Hypothetical protein B296_00005040  [Ensete ventricosum] | RRT85362.1 |
| 26 | PREDICTED: 36.4 kDa proline-rich protein-like  [Musa acuminata subsp. malaccensis] | XP_009380947.1 |
| 27 | Ammonium transporter 1 member 2-like  [Phoenix dactylifera] | XP_008801272.1 |
