# Supplementary material for: Screening and Identification of Host Proteins Interacting with Iris lactea var. chinensis Metallothionein IlMT2a by Yeast Two-Hybrid Assay
Source: Genes (Basel). 2021 Apr 10;12(4):554. doi: 10.3390/genes12040554 (PMC8069374; doi:10.3390/genes12040554)
Supplement: Supplementary file 1 [file genes-12-00554-s001.zip › Supplement/Table S2 The result of root lengths statistical an.docx]

**Table S2** The result of root lengths statistical analysis of t-test after Cd treatment.

|  | WT/OE3 | WT/OE4 | WT/OE7 |
| --- | --- | --- | --- |
| 1/2MS | 0.101191507 | 0.539079574 | 0.101191507 |
| 25uM CdCl | 0.205106455 | 0.144703999 | 0.094446327 |
| 50 CdCl | 0.205106455 | 1 | 0.25081536 |
| 1OO CdCl | 0.024110111 | 1 | 0.643329963 |
