# Supplementary figures and images for: Screening and Identification of Host Proteins Interacting with Iris lactea var. chinensis Metallothionein IlMT2a by Yeast Two-Hybrid Assay
Source: Genes (Basel). 2021 Apr 10;12(4):554. doi: 10.3390/genes12040554 (PMC8069374; doi:10.3390/genes12040554)

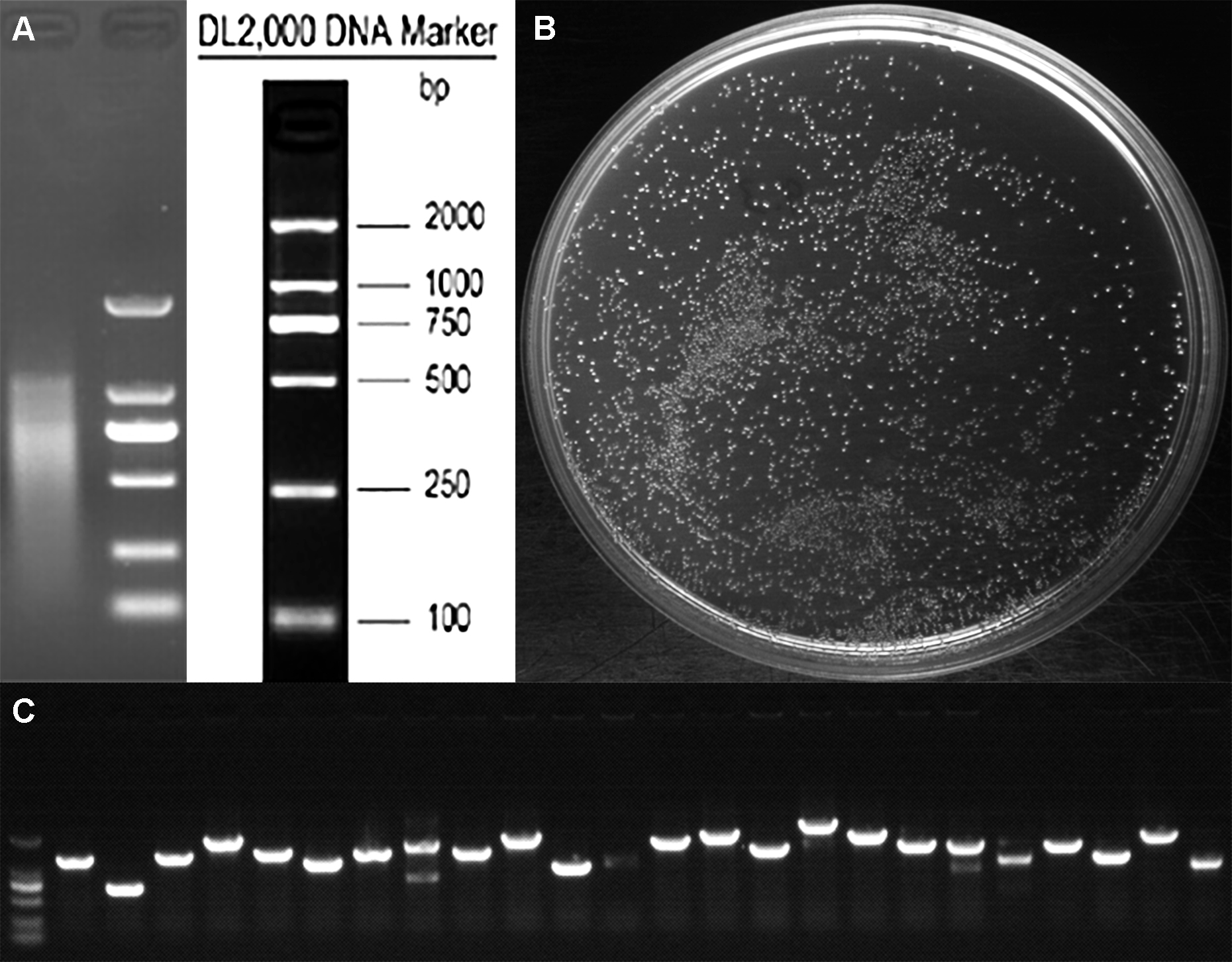

Supplement: Supplementary file 1 [file genes-12-00554-s001.zip › Supplement/Figure S1.jpg]

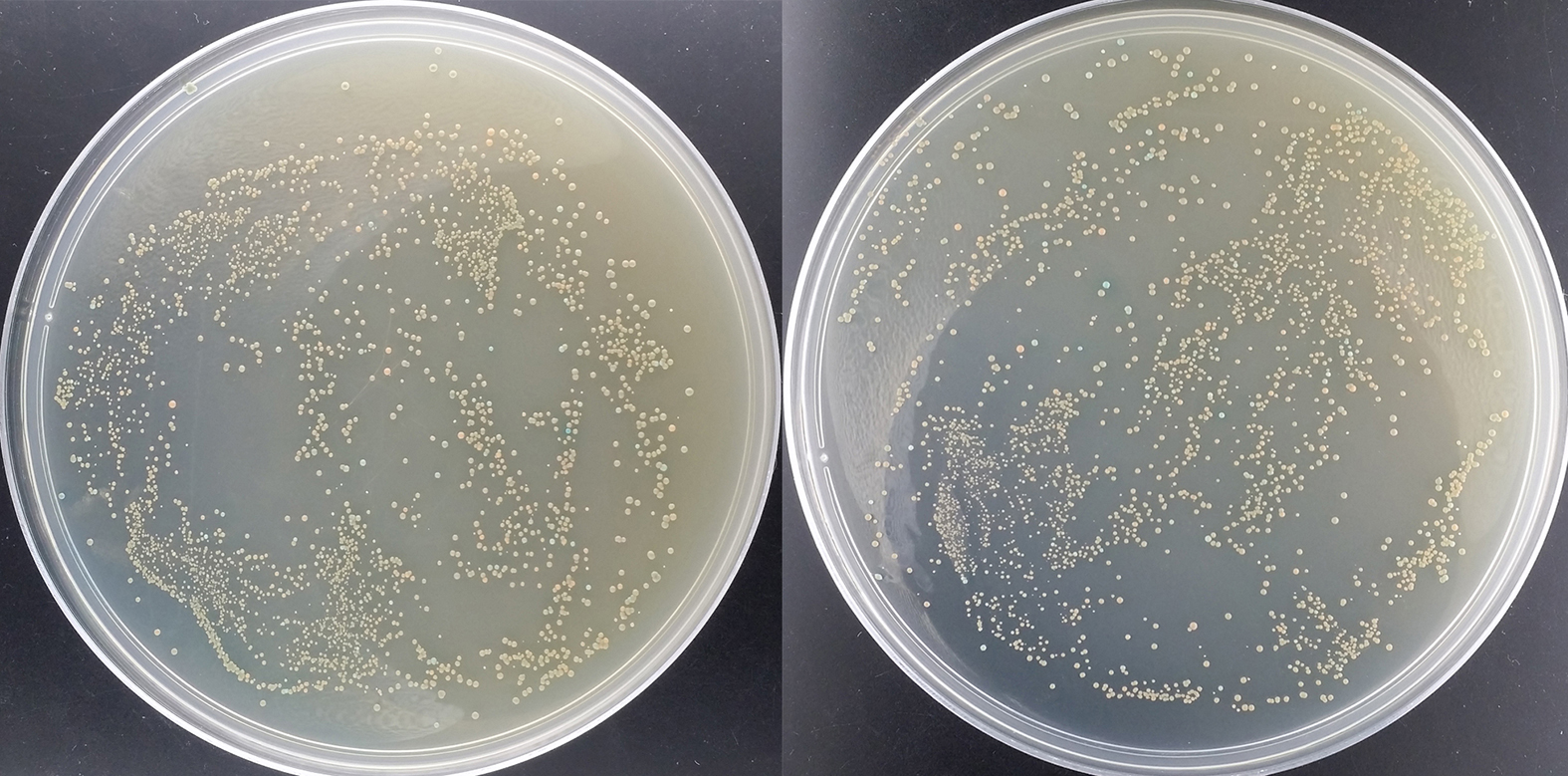

Supplement: Supplementary file 1 [file genes-12-00554-s001.zip › Supplement/Figure S2.jpg]

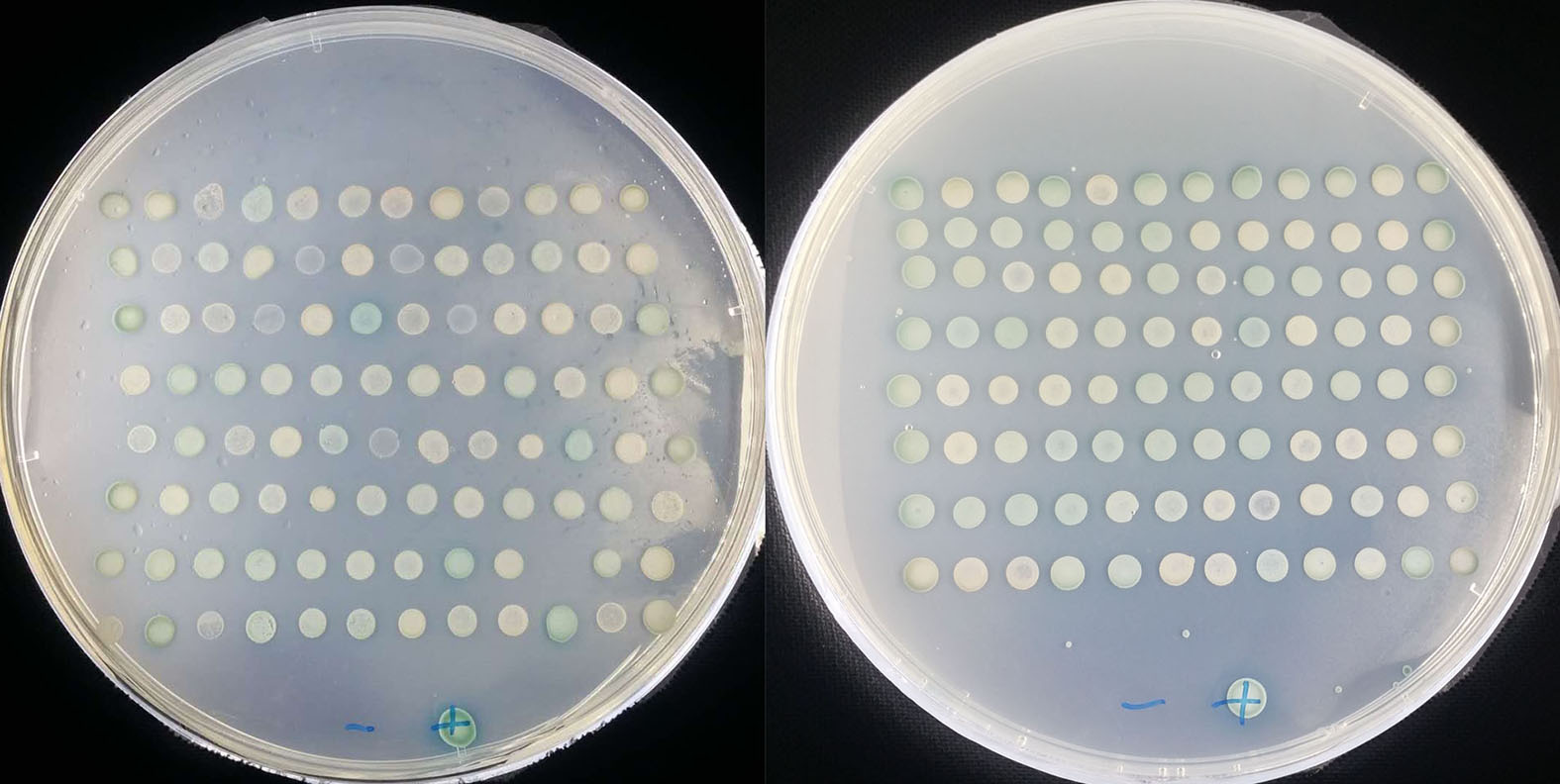

Supplement: Supplementary file 1 [file genes-12-00554-s001.zip › Supplement/Figure S3.jpg]

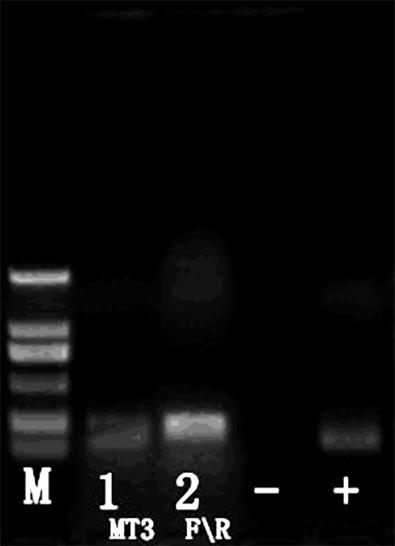

Supplement: Supplementary file 1 [file genes-12-00554-s001.zip › Supplement/Figure S4.png]
